# Increased artemisinin production by promoting glandular secretory trichome formation and reconstructing the artemisinin biosynthetic pathway in *Artemisia annua*

**DOI:** 10.1093/hr/uhad055

**Published:** 2023-03-28

**Authors:** Yongpeng Li, Wei Qin, Hang Liu, Tiantian Chen, Xin Yan, Weizhi He, Bowen Peng, Jin Shao, Xueqing Fu, Ling Li, Xiaolong Hao, Guoyin Kai, Kexuan Tang

**Affiliations:** Frontiers Science Center for Transformative Molecules, Joint International Research Laboratory of Metabolic & Developmental Sciences, Key Laboratory of Urban Agriculture (South) Ministry of Agriculture, Plant Biotechnology Research Center, Fudan-SJTU-Nottingham Plant Biotechnology R & D Center, School of Agriculture and Biology, Shanghai Jiao Tong University, Shanghai 200240, China; Laboratory of Medicinal Plant Biotechnology, School of Pharmaceutical Sciences, Academy of Chinese Medical Science, Zhejiang Chinese Medical University, Hangzhou 310053, China; Frontiers Science Center for Transformative Molecules, Joint International Research Laboratory of Metabolic & Developmental Sciences, Key Laboratory of Urban Agriculture (South) Ministry of Agriculture, Plant Biotechnology Research Center, Fudan-SJTU-Nottingham Plant Biotechnology R & D Center, School of Agriculture and Biology, Shanghai Jiao Tong University, Shanghai 200240, China; Frontiers Science Center for Transformative Molecules, Joint International Research Laboratory of Metabolic & Developmental Sciences, Key Laboratory of Urban Agriculture (South) Ministry of Agriculture, Plant Biotechnology Research Center, Fudan-SJTU-Nottingham Plant Biotechnology R & D Center, School of Agriculture and Biology, Shanghai Jiao Tong University, Shanghai 200240, China; Frontiers Science Center for Transformative Molecules, Joint International Research Laboratory of Metabolic & Developmental Sciences, Key Laboratory of Urban Agriculture (South) Ministry of Agriculture, Plant Biotechnology Research Center, Fudan-SJTU-Nottingham Plant Biotechnology R & D Center, School of Agriculture and Biology, Shanghai Jiao Tong University, Shanghai 200240, China; Frontiers Science Center for Transformative Molecules, Joint International Research Laboratory of Metabolic & Developmental Sciences, Key Laboratory of Urban Agriculture (South) Ministry of Agriculture, Plant Biotechnology Research Center, Fudan-SJTU-Nottingham Plant Biotechnology R & D Center, School of Agriculture and Biology, Shanghai Jiao Tong University, Shanghai 200240, China; Frontiers Science Center for Transformative Molecules, Joint International Research Laboratory of Metabolic & Developmental Sciences, Key Laboratory of Urban Agriculture (South) Ministry of Agriculture, Plant Biotechnology Research Center, Fudan-SJTU-Nottingham Plant Biotechnology R & D Center, School of Agriculture and Biology, Shanghai Jiao Tong University, Shanghai 200240, China; Frontiers Science Center for Transformative Molecules, Joint International Research Laboratory of Metabolic & Developmental Sciences, Key Laboratory of Urban Agriculture (South) Ministry of Agriculture, Plant Biotechnology Research Center, Fudan-SJTU-Nottingham Plant Biotechnology R & D Center, School of Agriculture and Biology, Shanghai Jiao Tong University, Shanghai 200240, China; Frontiers Science Center for Transformative Molecules, Joint International Research Laboratory of Metabolic & Developmental Sciences, Key Laboratory of Urban Agriculture (South) Ministry of Agriculture, Plant Biotechnology Research Center, Fudan-SJTU-Nottingham Plant Biotechnology R & D Center, School of Agriculture and Biology, Shanghai Jiao Tong University, Shanghai 200240, China; Frontiers Science Center for Transformative Molecules, Joint International Research Laboratory of Metabolic & Developmental Sciences, Key Laboratory of Urban Agriculture (South) Ministry of Agriculture, Plant Biotechnology Research Center, Fudan-SJTU-Nottingham Plant Biotechnology R & D Center, School of Agriculture and Biology, Shanghai Jiao Tong University, Shanghai 200240, China; Frontiers Science Center for Transformative Molecules, Joint International Research Laboratory of Metabolic & Developmental Sciences, Key Laboratory of Urban Agriculture (South) Ministry of Agriculture, Plant Biotechnology Research Center, Fudan-SJTU-Nottingham Plant Biotechnology R & D Center, School of Agriculture and Biology, Shanghai Jiao Tong University, Shanghai 200240, China; Laboratory of Medicinal Plant Biotechnology, School of Pharmaceutical Sciences, Academy of Chinese Medical Science, Zhejiang Chinese Medical University, Hangzhou 310053, China; Laboratory of Medicinal Plant Biotechnology, School of Pharmaceutical Sciences, Academy of Chinese Medical Science, Zhejiang Chinese Medical University, Hangzhou 310053, China; Frontiers Science Center for Transformative Molecules, Joint International Research Laboratory of Metabolic & Developmental Sciences, Key Laboratory of Urban Agriculture (South) Ministry of Agriculture, Plant Biotechnology Research Center, Fudan-SJTU-Nottingham Plant Biotechnology R & D Center, School of Agriculture and Biology, Shanghai Jiao Tong University, Shanghai 200240, China

Dear Editor,

Artemisinin, which has potent antimalarial properties, is a sesquiterpene endoperoxide originally isolated from the traditional Chinese medicinal plant *Artemisia annua*. However, the artemisinin content in wild-type (WT) *A. annua* is low (1–10 mg/g dry weight), leading to its erratic supply and price fluctuations [1]. Recent advances in synthetic biology tools have enabled researchers to engineer several species, including *Nicotiana benthamiana* and *Physcomitrella patens* to produce artemisinin [2, 3]. In *Saccharomyces cerevisiae*, only high-yielding production of the precursor of artemisinin, artemisinic acid (AA), has been achieved, while the synthesis of artemisinin still strictly depends on semi-chemical methods [4]. Meanwhile, current artemisinin production using heterologous expression systems is limited and far from meeting global demand, and *A. annua* remains its primary commercial source [5].

**Figure 1:**
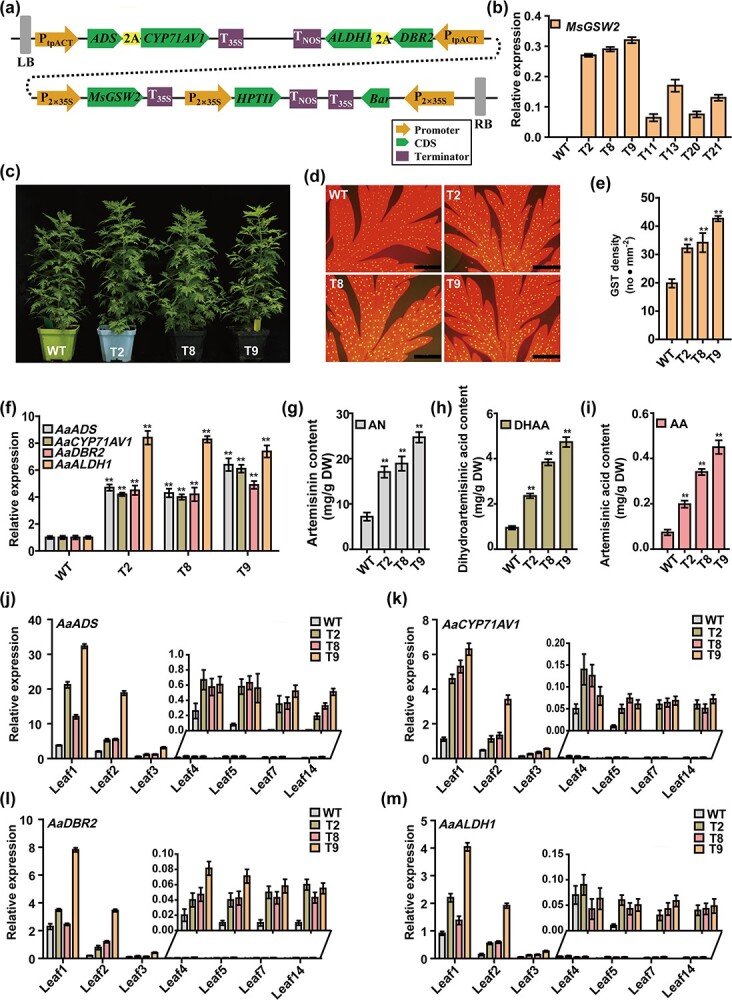
Increased artemisinin production using a synthetic biology strategy in *A. annua*. **a** Schematic illustrating the construct for artemisinin engineering in *A. annua*. **b** Relative expression levels of *MsGSW2* in leaf 0 of WT and transgenic *A. annua* lines determined by qPCR. *β-Actin* (EU531837.1) was used as the control to normalize gene expression with the 2^−ΔCT^ method. **c** Morphology of WT and transgenic *A. annua* plants. **d** Peltate GSTs on fully expanded leaves derived from WT and *T*_2_, *T*_8_, and *T*_9_ plants. Images were collected using fluorescence microscopy under a 4× objective. Yellow autofluorescence represents peltate GSTs and red autofluorescence represents chlorophyll. Scale bars = 1 mm. **e** Peltate GST densities of fully expanded leaves derived from WT and *T*_2_, *T*_8_, and *T*_9_ plants. **f** Relative expression levels of *AaADS*, *AaCYP71AV1*, *AaDBR2*, and *AaALDH1* in leaf 0 of WT and *T*_2_, *T*_8_, and *T*_9_ plants determined by qPCR. *β-Actin* was used to normalize gene expression with the 2^−ΔΔCT^ method. **g** Artemisinin (AN) content of WT and *T*_2_, *T*_8_, and *T*_9_ plants. **h** Dihydroartemisinic acid (DHAA) content of WT and *T*_2_, *T*_8_, and *T*_9_ plants. **i** Artemisinic acid (AA) content of WT and *T*_2_, *T*_8_, and *T*_9_ plants. **j**–**m** Relative expression levels of *AaADS*, *AaCYP71AV1*, *AaDBR2*, and *AaALDH1* in leaves at different positions of WT and *T*_2_, *T*_8_, and *T*_9_ plants determined by qPCR. *β-Actin* was used to normalize gene expression with the 2^−ΔCT^ method. Data values are presented as mean ±standard deviation (*n* = 3) of three replicates from three cutting propagations*.* Student’s *t*-test: ^**^P < .01.

Artemisinin is known to be phytotoxic, and its biosynthesis is confined to *A. annua* peltate glandular secretory trichomes (GSTs), which can specifically store artemisinin and surmount the problems caused by cellular toxicity [6]. The peltate GST density of *A. annua* is positively associated with artemisinin yield. For example, GST-specific WRKY transcription factor 2 from *A. annua* (AaGSW2) and its homologs in *Mentha haplocalyx* (MhGSW2) and *Mentha spicata* (MsGSW2) could significantly promote GST formation and lead to increased artemisinin content in transgenic *A. annua* plants by 56–100% [7]. Promoting GST formation in *A. annua* plants represents a promising strategy for artemisinin improvement [8]. It is also known that artemisinin biosynthetic genes such as *AaADS*, *AaCYP71AV1*, *AaDBR2*, and *AaALDH1* are specifically expressed in the peltate GSTs of young leaves, whereas their expression is almost undetectable in old leaves [6, 9], suggesting that the efficient synthesis of artemisinin mainly occurs in the immature peltate GSTs. Therefore, reconstructing the artemisinin biosynthetic pathway in the peltate GSTs of old leaves of *A. annua* has the potential to improve artemisinin biosynthesis. Given the advantages of the GoldenBraid cloning system, a multigene construct harboring seven genes, including *MsGSW2*, which promotes peltate GST initiation, and four core artemisinin biosynthetic genes, was generated (Fig. 1a). To overexpress *MsGSW2*, it was introduced under the control of the 2X35S promoter. Since the currently identified *A. annua* GST-specific promoters and 35S promoter are always inactive in the peltate GSTs of old leaves, the tpACT promoter, which has proved to be active in the peltate GSTs of both young and old leaves, was used to drive the expression of artemisinin biosynthetic genes [9]. In addition, *AaADS* and *AaCYP71AV1*, as well as *AaDBR2* and *AaALDH1*, were respectively linked by a cleavable 2A peptide [10], which allows reduced usage of promoters (Fig. 1a).

As illustrated in Fig. 1b, the expression levels of *MsGSW2* in the generated transgenic *A. annua* plants harboring the seven-gene construct were uneven, which might result from the location of transferred genes or the corresponding epigenetic regulation of these genes. Three independent transgenic lines (*T*_2_, *T*_8_, and *T*_9_) that showed higher *MsGSW2* expression level were then chosen for further analysis. No obvious morphological differences were observed between the WT and transgenic *A. annua* plants (Fig. 1c). The GST density on the leaves of transgenic *A. annua* plants was substantially stimulated, with an increase of 1.7- to 2.2-fold (Fig. 1d and e). Generally, the artemisinin biosynthetic genes, including *AaADS*, *AaCYP71AV1*, *AaDBR2*, and *AaALDH1*, show highest expression in leaf 0 (shoot). We firstly analyzed the expression levels of these genes in leaf 0 of WT and transgenic plants. As expected, they were significantly increased in leaves of *T*_2_, *T*_8_, and *T*_9_ plants in comparison with WT (Fig. 1f). Correspondingly, the transgenic *A. annua* plants produced significantly higher (2.4- to 3.4-fold) amounts of artemisinin than those in WT plants, as measured by high-performance liquid chromatography (HPLC) analysis (Fig. 1g). Notably, the transgenic *A. annua* plants obtained in this study showed increased artemisinin production compared with both WT and plants overexpressing only *MsGSW2* [7], indicating that the strategy of increasing the GST density as well as the expression levels of artemisinin biosynthetic genes had a cumulative effect on artemisinin yield. Among the transgenic lines, line *T*_9_ produced the highest artemisinin content (24.7 mg/g dry weight). In addition, the contents of the two precursors of artemisinin, AA and dihydroartemisinic acid (DHAA), in transgenic *A. annua* plants were also significantly increased (Fig. 1h and i). Since the tpACT promoter is consistently active in the leaves at different positions of *A. annua* plants, the expression levels of artemisinin biosynthetic genes in leaves at different positions (leaves 1, 2, 3, 4, 5, 7, and 14) were analyzed. In WT plants, the expression levels of *AaADS*, *AaCYP71AV1*, *AaDBR2*, and *AaALDH1* reduced sharply in old leaves (leaves 4, 5, 7, and 14), whereas higher expression levels were observed in those of transgenic plants, demonstrating the potential application of the tpACT promoter in *A. annua* research (Fig. 1j–m). In summary, successful enhancement of artemisinin in *A. annua* using plant synthetic biology tools was achieved. The results presented in this study provide hope for remarkable improvement of artemisinin production, as well as the synthesis of other high-value natural products in *A. annua* plants, taking advantage of GSTs as natural cell factories.

## Acknowledgements

This work was supported by National Key R&D Program of China (2018YFA0900600), the Bill & Melinda Gates Foundation (OPP1199872 and INV-027291), the China Postdoctoral Science Foundation (2022M722851), the National Natural Science Foundation of China (82274047, 31770327, 32070329, 82003889), SJTU Trans-med Awards Research (20190104), SJTU Global Strategic Partnership Fund (2020 SJTU-CORNELL), Zhejiang Provincial Natural Science Foundation of China (LQ21H280004), National Young Qihuang Scholars Training Program, and the National ‘Ten-thousand Talents Program’ for Leading Talents of Science and Technology Innovation in China.

## Author contributions

Y.L. and K.T. designed the research. Y.L., W.Q., H.L., T.C., X.Y., W.H., B.P., and J.S. performed most of the experiments. Y.L. drafted the manuscript. Y.L., W.Q., X.F., L.L., X.H., G.K., and K.T. revised the manuscript. All authors have approved the manuscript.

## Data availability

All data supporting this study are available in the article.

## Conflict of interest

The authors declare no conflicts of interest.
